# Application of the Neuroform Atlas Stent for Intracranial Stenting of Symptomatic Intracranial Atherosclerotic Stenosis: A Multi‐Center Retrospective Study

**DOI:** 10.1002/brb3.70798

**Published:** 2025-09-01

**Authors:** Weicheng Peng, Haiyang Ma, Shuaibin Lu, Xinli Xiang, Rui Zhao, Sheng Xu, Xupeng Peng, Yuhua Jiang, Zhiqiang Hu, Feng Guan

**Affiliations:** ^1^ Department of Neurosurgery Beijing Shijitan Hospital, Capital Medical University Beijing China; ^2^ Department of Neurosurgery Third Affiliated Hospital of Sun Yat‐Sen University Guangzhou Guangdong China; ^3^ Department of Pharmacy, Beijing Shijitan Hospital Capital Medical University Beijing China; ^4^ Department of Neurosurgery, Beijing Neurosurgical Institute, Beijing Tiantan Hospital Capital Medical University Beijing China

**Keywords:** cerebrovascular disorders, intracranial atherosclerotic stenosis, intracranial stenting, ischemic stroke, Neuroform Atlas stent, treatment

## Abstract

**Background:**

Intracranial stenting with the Neuroform Atlas stent is an emerging treatment option for symptomatic intracranial atherosclerotic stenosis. Nevertheless, the efficacy and safety of the Neuroform Atlas stent as an option for intracranial stenting remain debatable.

**Methods:**

This study enrolled clinical data from 264 consecutive patients diagnosed with symptomatic intracranial atherosclerotic stenosis treated with intracranial stenting with the Neuroform Atlas stent between January 2020 and February 2023 to assess the efficacy and safety of the procedure. The stenosis rate of the target artery was assessed using digital subtraction angiography, and the outcome of patients was evaluated using the modified Rankin Scale (mRS).

**Results:**

Among 264 patients, the mean stenosis rate of the target artery was 88.82% ± 6.35% before the procedure (T0), 47.99% ± 9.37% at the end of the procedure (T1), and 41.86% ± 7.30% at 6‐month follow‐up (T2). The stenosis rate was statistically significant between T0 and T1 (*p* = 0.00), between T0 and T2 (*p* = 0.00), and between T1 and T2 (*p* = 0.00). At 12 months postoperatively, 226 patients had a good outcome (mRS 0–2) without stroke recurrence attributed to the target artery, and 32 patients had a good outcome but with stroke recurrence. There were three cases of ischemic stroke and two cases of hemorrhagic stroke related to the stenting process. No intraprocedural deaths were reported.

**Conclusion:**

Intracranial stenting with the Neuroform Atlas stent is a potentially safe and effective treatment for symptomatic intracranial atherosclerotic stenosis. It demonstrates a statistically significant difference in the caliber of the target artery before and after treatment as well as significantly improves the cerebral ischemic symptoms of patients.

## Graphical abstract

Intracranial stenting with the Neuroform Atlas stent is a potentially safe and effective treatment for symptomatic intracranial atherosclerotic stenosis (sICAS). It demonstrates a statistically significant difference in the caliber of the target artery before and after treatment as well as significantly improves the cerebral ischemic symptoms of patients.

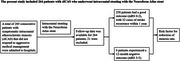

## 1 Introduction

Stroke is the second leading cause of death globally and the leading cause of mortality and disability rates in China, with ischemic stroke (IS) being the most prevalent stroke type (GBD 2017 Causes of Death Collaborators^2018^; Zhou et al. 2016). The burden is anticipated to rise due to an aging population, continued high prevalence of risk factors (e.g., hypertension), and inadequate treatment (Zhang et al. 2024). Specifically, recent data suggest that the age‐standardized incidence of acute ischemic stroke (AIS) ranges from 97.1 to 127.3 per 100,000 person‐years, with mortality to incidence ratio standing at 0.26–0.29 (Elmadhoun, Wang, and Ding 2024; Krishnamurthi et al. 2013). Additionally, intracranial atherosclerotic stenosis (ICAS) has been identified as a key factor of IS. In recent years, 31% of adults with typical cardiovascular risk factors were found to have ICAS, and 9% had stenosis levels more than 50%. Suri et al. (2016) found that 4.2% of patients with ICAS had a stenosis rate of 50%–69%, 3.1% had a stenosis rate of 70%–99%, and 1.7% were fully occluded.

Patients with ICAS caused by atherosclerotic plaques are at high risk of recurrent ischemic events. Despite appropriate medical treatment, 38.2% of patients experience a new cerebrovascular event within 2 years, among which 13.7% are AIS and 24.5% are transient ischemic attack (TIA) (Mazighi et al. 2006; Zhao et al. 2024). Recent studies from the WEAVE (Wingspan Stent System Post Market Surveillance)/WOVEN (Wingspan One‐year Vascular Events and Neurologic Outcomes) trial have demonstrated that intracranial stenting is a successful technique that prevents stroke recurrence in patients with symptomatic ICAS (sICAS) who have not responded to aggressive medical management (Alexander et al. 2019, 2021). Traditional intracranial arterial stenting systems are highly developed for use in endovascular therapy of ICAS; however, these stenting systems have shortcomings when targeting smaller responsible arteries. For instance, previous deployment of intracranial arterial stents required coaxial microcatheter exchange, which increased the risk of stenting failure and complications.

Stenting in small arteries has proven to be technically problematic due to the challenge of inserting larger (0.021 or 0.027 inch) delivery microcatheters into these small arteries. To improve the accessibility and success rate of the procedure, low‐profile stents that can be delivered through 0.0165‐ or 0.017‐inch microcatheters have recently been introduced, allowing access to narrower and more tortuous arteries (Santillan et al. 2018; Wang et al. 2017). Although low‐profile stents are required to treat aneurysms arising from a parent artery with a diameter of either ≥2.0 or ≥2.5 mm, their use for treating sICAS has been gradually acknowledged and supported by the relevant literature (Park et al. 2017; Zaidat et al. 2020). Notably, the Neuroform Atlas Stent System was not initially designed to treat sICAS, but as a low‐profile stent, it possesses technical features that make it suitable for this purpose (Buonomo et al. 2021; Ellenbogen et al. 2023). In our multi‐center study, we sought to verify the safety and efficacy of Neuroform Atlas stent for the stenting of 264 patients with sICAS.

## 2 Patients and Methods

### 2.1 Patient Recruitment

The inclusion criteria included (1) age >18 years; (2) ICAS (stenosis rate >70%) was confirmed preoperatively in single‐vessel, including intracranial internal carotid artery (ICA), M1 segment of middle cerebral artery (MCA), basilar artery (BA), and intracranial vertebral artery (VA); (3) patients with recurrent IS or TIA within the past 90 days due to hypoperfusion in the territory of the target lesion despite aggressive medical management, where IS was defined as new focal neurological deficit lasting ≥24 h or < 24 h with new infarction on imaging, and TIA was defined as acute onset of focal neurological deficit lasting <24 h without new infarction on imaging; (4) patients with significant hypoperfusion manifestations in brain tissue supplied by target artery visible on computed tomography perfusion (CTP) (A cerebral blood flow decrease of ≥30% when compared to the perfusion on the contralateral side for an anterior circulation lesion or the anterior circulation territory for a posterior circulation lesion) (Miao et al. 2015); (5) all patients underwent intracranial stenting by the same team of interventionalists at Beijing Shijitan Hospital or Beijing Tiantan Hospital.

The exclusion criteria included (1) patients with massive stroke in the past 30 days; (2) patients with a side branch neoanastomotic vessel on computed tomography angiography (CTA) or digital subtraction angiography (DSA), and no hypoperfusion on CTP (Yang et al. 2024); (3) patients presenting with cerebral infarction combined with cerebral hemorrhage; (4) nonatherosclerotic stenosis including moyamoya disease, muscle fiber dysplasia, or arterial dissection; (5) patients with serious diseases who could not tolerate anesthesia and surgery.

### 2.2 Clinical Data

After hospital admission, all patients underwent computed tomography (CT) and magnetic resonance imaging (MRI) (including DWI, T2, T1, and FLAIR sequences). Thereafter, CTA and CTP were performed to assess the extent of intracranial and extracranial vascular patency and perfusion of all brain tissues. Besides, vessel wall high‐resolution magnetic resonance imaging (VW‐HRMRI) was performed in patients' target artery to assess the condition of the vascular lumen. Plaque radiological features, including plaque burden, plaque thickness, and enhancement ratio, were extracted from VW‐HRMRI. The stenosis rate of the target artery was described by DSA and computed based on the following formulas: The stenosis rate = 1 − (*D*s/*D*n), where *D*s was the diameter of the artery where the stenosis was most obvious and *D*n was the diameter of the proximal normal artery (Samuels et al. 2000). We assessed the stenosis rate of the ICAS by DSA before and in the immediate postoperative period. The stenosis rates at these two time points were referred to as “T0” and “T1” in our study. Laboratory tests included routine blood counts, platelet aggregation tests, thrombelastograms, and genetic testing for clopidogrel resistance. Clinical assessments included the mRS before and after intracranial stenting.

### 2.3 Endovascular Procedure

All endovascular procedures were performed at Beijing Shijitan Hospital and Beijing Tiantan Hospital under general anesthesia by two senior neurointerventionalists with more than 10 years of experience in intracranial stenting. During the procedure, the anesthetist kept the systolic pressure of the patient under strict control at 90–140 mmHg, to prevent large fluctuations in blood pressure and heart rate. All patients underwent the creation of a right femoral artery working channel using the Seldinger technique, followed by a complete cerebral angiography using a 5F contrast catheter to define the responsible vessel and examine the stenosis rate in the responsible vessel.

In patients with a sICAS of the MCA, for example, the intraoperative access catheter was superselected to the distal C1 segment of the ICA, and the intermediate catheter was delivered coaxially to the C4 segment. The microguidewire discreetly advanced the microcatheter through the stenotic segment of the MCA, and the coaxial delivery of the microcatheter crossed the stenotic segment. Intraoperative microcatheterography revealed that the microcatheter was in the true lumen and a long microcatheter wire was replaced. The microcatheter wire coaxially delivered the Gateway balloon to the stenotic segment slowly inflated the balloon to six atmospheres and held for 15 s before slowly releasing the balloon. Imaging revealed stenosis dilatation, internal wall roughness, and a visible residual stenosis rate. Upon 10 min of observation and re‐imaging, we observed residual stenosis in the stenotic segment. The microcatheter was advanced coaxially along the microguidewire to the distal end of the stenotic segment. The Neuroform Atlas stent was released against the medial wall of the stenotic segment and the stent opened well with good apposition. Final imaging showed residual stenosis. All patients were sutured to the puncture site using an Abbott Vascular Suture Device; a balance was achieved by freehand and sandbag compressions.

### 2.4 Materials

The Neuroform Atlas stent (Stryker Neurovascular, Kalamazoo, MI, USA) is a low‐profile, open‐cell stent with a hybrid design that can be delivered via a 0.0165‐ or 0.017‐inch catheter. The structure of the Neuroform Atlas stent is an alternating crown of eight and 12 struts. The eight‐strut crown has a thicker wire to ensure optimal apposition to the vessel wall. The 12 strut crown has a thinner wire to optimize stent conformability. The alternation of open and closed cell structures makes the Neuroform Atlas stent a hybrid design stent. The Neuroform Atlas stent is a fluoroscopically invisible nickel‐titanium alloy stent with three proximal and distal radiopaque markers. During deployment, the stent maintains a detectable length within the microcatheter with little or no premature shortening, providing high landing accuracy (Zaidat et al. 2020). It is suited for stent catheters with diameters between 2 and 4.5 mm. The available stent diameters range from 3 to 4.5 mm, with stent lengths of 15, 21, 24, and 30 mm (Stracke et al. 2020). We initially chose the Gateway for the PTA balloon, with the Neurospeed (Acandis, Pforzheim, Germany) as an alternative. The balloon is delivered via the Envoy guide catheter (Codman Neurovascular, Raynham, MA, USA), and the stent is released using either the Echelon 10 microcatheter (ev3 Endovascular, Inc., Plymouth, MN, USA) or the Excelsior SL‐10 microcatheter (Stryker, Fremont, CA, USA). The size selection of the angioplasty device is based on the intrinsic diameter of the target vessel and the length of the stenotic lesion.

### 2.5 Evaluation and Medical Therapy

Before the intracranial stenting procedure, each patient received dual antiplatelet medication with aspirin (100 mg once daily) and clopidogrel (75 mg once daily). Patients who were hyporesponders to clopidogrel received additional ticagrelor (90 mg twice daily). After the intracranial stenting procedure, all patients were evaluated by DSA after the Neuroform Atlas stent was confirmed to be morphologically stable. All patients were intensively monitored in the ICU postoperatively until transfer to the general ward after dressing removal; the patients then received optimal pharmacological treatment according to comorbidities. Aspirin (100 mg once daily) plus clopidogrel (75 mg once daily) or ticagrelor (90 mg twice daily) were prescribed after intracranial stenting; however, clopidogrel or ticagrelor would be discontinued 90 days after the intracranial stenting process based on the antiplatelet drug resistance test.

### 2.6 Radiologic and Clinical Follow‐up

After the intracranial stenting procedure, all patients underwent a neurological examination (including assessment of mRS). All patients returned to the hospital for DSA at 6 months postoperatively. The stenosis rate at this time point was referred to as “T2.” We assessed 100% of the canal lumen, and the stenosis rate at each time point was assessed by three experienced neurointerventionalists, selecting the average of the ICAS measurements at each time point. Clinical status at follow‐up was assessed by experienced senior interventionalists and neuroimaging physicians who jointly assessed clinical and imaging data.

The criteria necessary for stroke recurrence diagnosis included (1) a sudden onset of fresh focal neurological deficits in the area previously affected, with symptoms lasting longer than 24 h and clear imaging evidence; (2) more than two TIA symptoms with clear distribution in the area of the ICAS, even in the absence of clear imaging evidence of stroke; (3) the exclusion of tumor, cerebral hemorrhage, among other causes (Coull and Rothwell 2004).

### 2.7 Statistical Analysis

All data were analyzed using statistical software (SPSS version 25.0; IBM Corp, Armonk, NY, USA). Continuous variables were presented as mean ± standard deviation. Count variables were presented as frequency (percentage). A paired‐sample *t*‐test was used to evaluate whether intracranial stenting with the Neuroform Atlas stent significantly reduced the stenosis rate of ICAS between different time points (T0 vs. T1; T0 vs. T2; T1 vs. T2) and relieved ischemic symptoms (mRS‐pre vs mRS‐post). Univariate and multivariate linear regression analyses were used to evaluate the stenosis rate between time points for specific patient characteristics (age, sex, hypertension, dyslipidemia, diabetes, smoking exposure, alcoholism, target artery, and qualifying ischemic events). A value of *p* < 0.05 was considered statistically significant.

## 3 Results

### 3.1 Clinical Data

**FIGURE 1:**
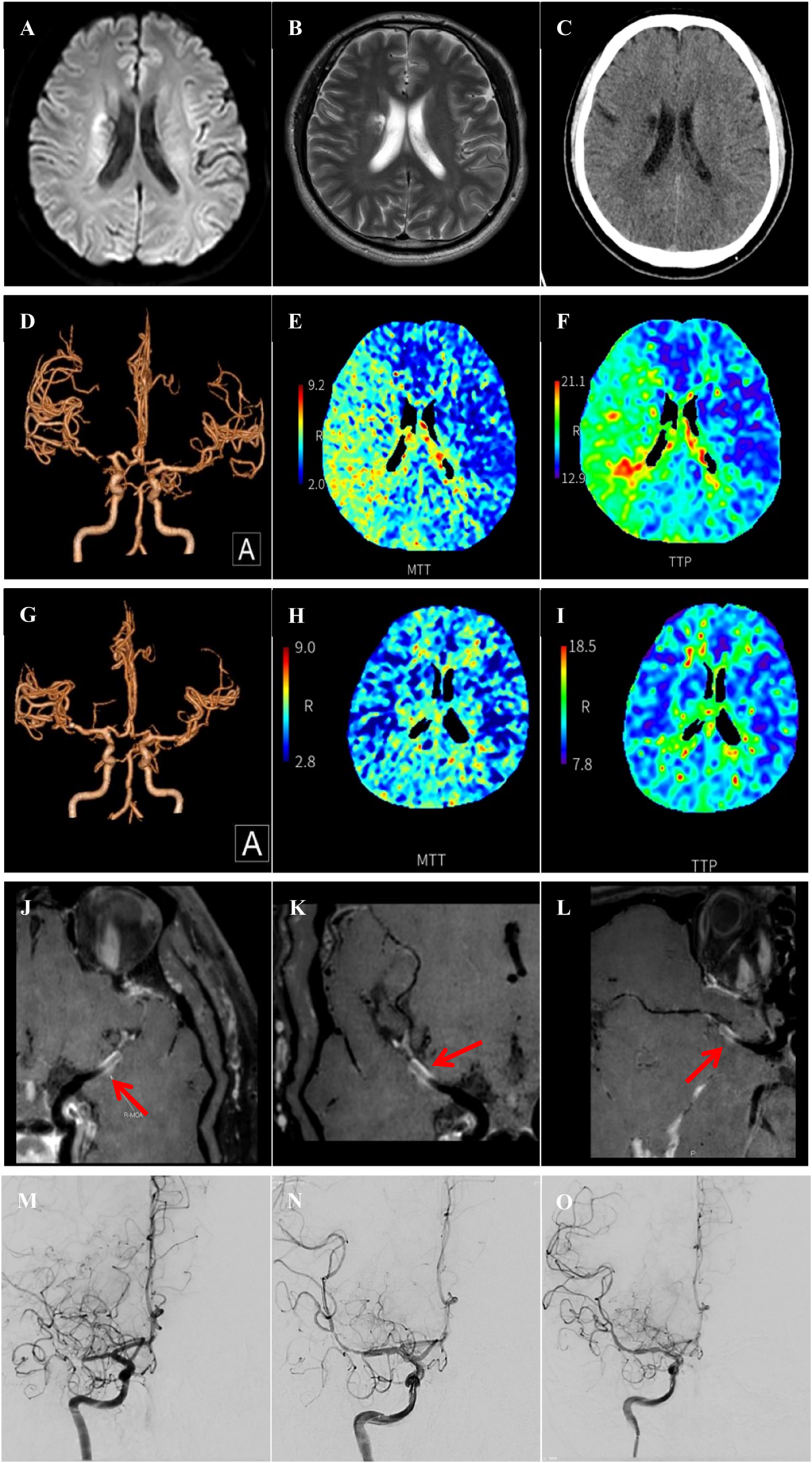
A case of sICAS in right MCA. (A–C): New infarction of right paraventricular white matter was observed in MRI and CT. (D–F): Preoperative CTA and CTP showed that M1 of the right MCA was nearly occluded and the brain tissue it innervates was hypoperfused. (G–I): Postoperative CTA and CTP showed that almost normal blood flow was restored to the M1 of the right MCA, and the corresponding cerebral tissue hypoperfusion was effectively improved. (J–L): Preoperative VW‐HRMRI indicated that the lumen of M1 of the right MCA was almost occluded, but a portion of the true lumen of the vessel wall remained, making it a candidate for angioplasty and stenting. (M–O): Preoperative DSA showed that the lumen of M1 of the right MCA was nearly occluded, and the distal branches could not be visualized. After angioplasty, a Neuroform Atlas stent was successfully implanted in the M1 of the right MCA, and follow‐up angiography 10 min later showed good blood flow. CT, computed tomography; CTA, computed tomography angiography; CTP, computed tomography perfusion; DSA, digital subtraction angiography; MCA, middle cerebral artery; MRI, magnetic resonance imaging; sICAS, symptomatic intracranial atherosclerotic stenosis; VW‐HRMRI, vessel wall high‐resolution magnetic resonance imaging.

**FIGURE 2:**
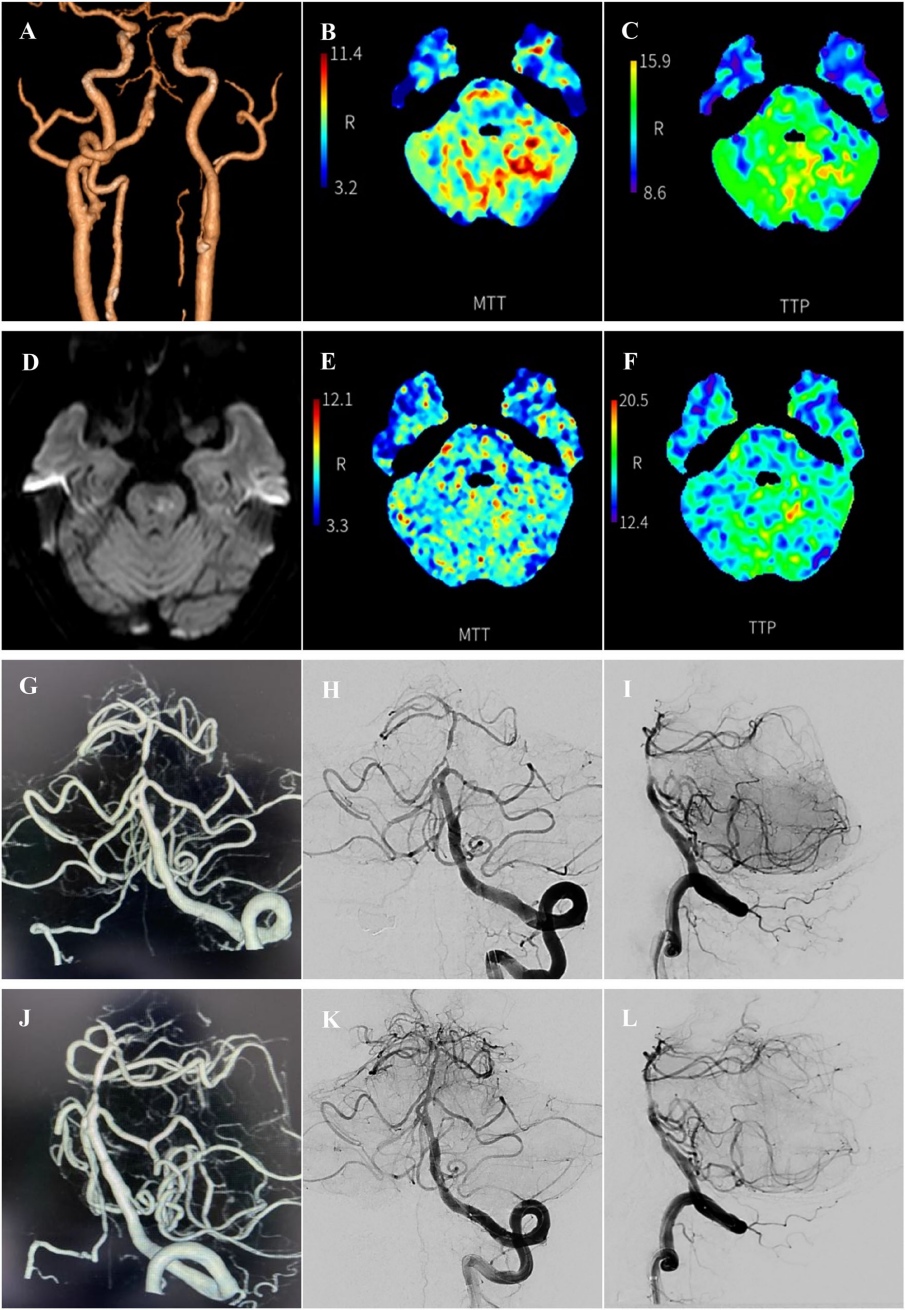
A case of sICAS in BA. (A–C): Preoperative CTA and CTP revealed severe stenosis of BA and the brain tissue it innervates was hypoperfused. (D): New infarction of brainstem was observed in the MRI. (E and F): Postoperative CTP showed that the corresponding cerebral tissue hypoperfusion was significantly improved. (G–J): Preoperative DSA showed severe stenosis of the lumen of BA, and the distal branches were sparse. (K and L): After angioplasty, a Neuroform Atlas stent was successfully implanted in the BA, and follow‐up angiography 10 min later showed good blood flow. BA, basilar artery; CTA, computed tomography angiography; CTP, computed tomography perfusion; DSA, digital subtraction angiography; MRI, magnetic resonance imaging; sICAS, symptomatic intracranial atherosclerotic stenosis.

**FIGURE 3:**
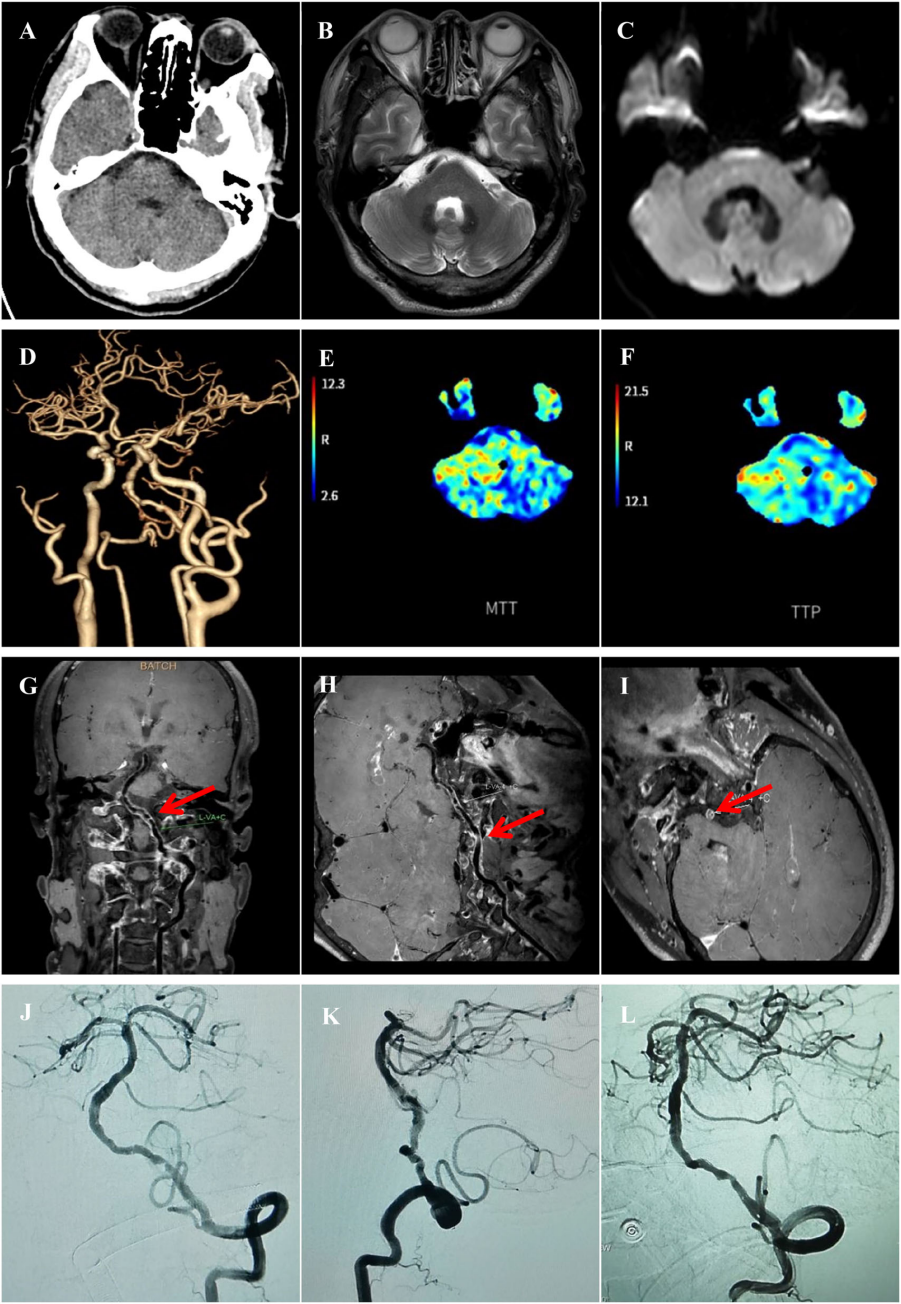
A case of sICAS in left VA. (A–C): Sporadic new infarction of pons was observed in the MRI and CT. (D–F): Preoperative CTA and CTP indicated severe stenosis of the V4 of left VA and the brain tissue it innervates was hypoperfused. (G–I): Preoperative VW‐HRMRI showed that the lumen of V4 of the left VA was almost occluded, but a portion of the true lumen of the vessel wall remained, making it a candidate for angioplasty and stenting. (J and K): Preoperative DSA showed severe stenosis of the lumen of VA, and the distal branches were sparse. (L): After angioplasty, a Neuroform Atlas stent was successfully implanted in the V4 of left VA, and follow‐up angiography 10 min later showed good blood flow. CT, computed tomography; CTA, computed tomography angiography; CTP, computed tomography perfusion; DSA, digital subtraction angiography; MRI, magnetic resonance imaging; sICAS, symptomatic intracranial atherosclerotic stenosis; VA, vertebral artery; VW‐HRMRI, vessel wall high‐resolution magnetic resonance imaging.

A total of 285 consecutive patients with sICAS who underwent intracranial stenting with the Neuroform Atlas stent were screened at Beijing Shijitan Hospital and Beijing Tiantan Hospital between January 2020 and February 2023. In total, 264 cases (mean age 62.71 ± 11.10 years, 62.12% males) were ultimately eligible for our study, followed up using DSA. Among the 21 excluded patients, 18 patients did not return to the hospital for DSA review, three patients died of other systemic diseases within 12 months of intracranial stenting, and no patients died of intracranial stenting or IS. Risk factors for sICAS included hypertension in 202 (202/264, 76.52%), dyslipidemia in 217 (217/264, 82.20%), diabetes in 160 (160/264, 60.61%), smoking in 140 (140/264, 53.03%, 104 cases of current smoker, 36 cases of previous smokers), and alcoholism in 121 (121/264, 45.83%, 86 cases of current drinkers, 35 cases of previous drinkers). There were 10 cases of sICAS of ICA, 109 of MCA, 82 of BA, and 63 of VA. For the qualifying ischemic event, 189 patients with IS (189/264, 71.59%) and 75 patients with TIA (75/264, 28.41%). For pre‐hospital antiplatelet medication, 225 (225/264, 85.23%) cases had dual antiplatelet medication with aspirin (100 mg once daily) and clopidogrel (75 mg once daily) and 39 (39/264, 14.77%) cases had aspirin (100 mg once daily) and ticagrelor (90 mg twice daily). Balloon angioplasty before stenting was indicated in 264 (264/264, 100.00%) cases and post‐stenting angioplasty in three (3/264, 1.14%) cases. We have only used the Gateway in 264 patients, with no patients using the Neurospeed as an alternative balloon. Baseline characteristics are shown in Table 1, and pre‐ and postoperative imaging and intraoperative cerebrovascular angiography of typical cases are shown in figure legends (Figures 1, 2, 3).

**TABLE 1:** Baseline characteristics of patients (N = 264).

Variables	Values
Mean age ± SD, years	62.71 ± 11.10
Males, *n* (%)	164 (62.12)
Hypertension, *n* (%)	202 (76.52)
Dyslipidemia, *n* (%)	217 (82.20)
Diabetes, *n* (%)	160 (60.61)
Smoking exposure, *n* (%)	
Current smoker	104 (39.39)
Previous smoker	36 (13.64)
Never smoked	124 (46.97)
Alcoholism, *n* (%)	
Current drinker	86 (32.58)
Previous drinker	35 (13.25)
Never drank	143 (54.17)
Target artery, *n* (%)	
ICA	10 (3.79)
MCA	109 (41.29)
BA	82 (31.06)
VA	63 (23.86)
Qualifying ischemic events, *n* (%)	
IS	189 (71.59)
TIA	75 (28.41)
Antiplatelet	
Aspirin and clopidogrel	225 (85.23)
Aspirin and ticagrelor	39 (14.77)
Angioplasty before stent	264 (100.00)
Angioplasty after stent	3 (1.14)

Abbreviations: BA: basilar artery; ICA: internal carotid artery; IS: ischemic stroke; MCA: middle cerebral artery; TIA: transient ischemic attacks; VA: vertebral artery.

### 3.2 Early and Long‐Term Outcome

The success of the intracranial stenting procedure was achieved in 100% (285/285) of treated patients. A total of 264 patients completed the radiological follow‐up (DSA) at 6 months after intracranial stenting. At the 12‐month postoperative follow‐up, 226 patients had a good outcome (mRS 0–2) without stroke recurrence attributed to the target artery, 32 patients had a good outcome (mRS 0–2) but with stroke recurrence in the same territory; six patients experienced a 12‐month negative outcome (mRS 3–5). A total of 79 (79/264, 29.92%) patients had mRS 0–2, and 185 (185/264, 71.08%) patients had mRS 3–5 in our population before intracranial stenting. At 12 months follow‐up after intracranial stenting, 258 (258/264, 97.73%) patients had mRS 0–2, and 6 (6/264, 2.27%) patients had mRS 3–5. The paired‐sample *t*‐test between pre‐ and post‐operative showed a statistically significant difference (*p* = 0.032). We noted three cases of ischemic stroke and two cases of hemorrhagic stroke related to the intracranial stenting process, two cases of transient central facial paralysis and five cases of transient limb numbness, and six cases of transient dysphagia. No intraprocedural deaths were reported, and neither did any patient undergo subsequent endovascular treatment after intracranial stenting. In this study, intracranial stenting with the Neuroform Atlas stent for sICAS had an overall complication rate of 6.06% (16/264), a 6‐month in‐stent restenosis (ISR) rate (stenosis rate ≥70%) of 1.89% (5/264), a stroke recurrence rate of 12.12% (32/264), and no deaths (Table 2).

**TABLE 2:** Complications of sICAS patients after intracranial stenting with the Neuroform Atlas stent (N=264).

Complications	No. of patients (%)
New infarction	3 (1.14)
Intracerebral hemorrhage	2 (0.76)
Transient central facial paralysis	2 (0.76)
Transient limb numbness	5 (1.89)
Transient dysphagia	6 (2.27)
12‐Months mortality	0 (0)
Stent occlusion or in‐stent restenosis	5 (1.89)
Arterial dissection	0 (0)
Vessel perforation	0 (0)

Abbreviation: sICAS: symptomatic intracranial atherosclerotic stenosis.

### 3.3 Reduction of Stenosis Rate

The mean length of the ICAS was 7.43 ± 6.21 mm (range 2.5–16.2 mm). We noticed a good improvement in target artery caliber with restoration of the stenotic lumen compared to preoperative conditions. The mean stenosis rate of ICAS was 88.82% ± 6.35% before surgery (T0), 47.99% ± 9.37% at the end of surgery (T1), and 41.86% ± 7.30% at the time of follow‐up (T2). As mentioned above, the mean reduction of stenosis rate was 40.83% ± 11.37% from T0 to T1, 6.13% ± 2.65% from T1 to T2, and 46.96% ± 9.81% from T0 to T2 (Table 3). Paired‐sample *t*‐test showed that the stenosis rate of ICAS was statistically significant between T0 and T1 (*p* = 0.00), T0 and T2 (*p* = 0.00), as well as T1 and T2 (*p* = 0.00).

**TABLE 3:** Procedural outcomes and paired‐sample *t*‐test (N = 264).

Pre/post‐treatment data	Values (mean ± sd)	*t* value	*p* value
ICAS length (mm)	7.43 ± 6.21	—	—
Stenosis rate			
Stenosis rate T0 (%)	88.82 ± 6.35	—	—
Stenosis rate T1 (%)	47.99 ± 9.37	—	—
Stenosis rate T2 (%)	41.86 ± 7.30	—	—
Percentage reduction			
Percentage reduction T0–T1 (↓%)	40.83± 11.37	—	—
Percentage reduction T1–T2 (↓%)	6.13 ± 2.65	—	—
Percentage reduction T0–T2 (↓%)	46.96 ± 9.81	—	—
Percentage of patients with an mRS 0–2			
Pre‐stenting	29.92% (79/264)	—	—
Post‐stenting	97.73% (258/264)	—	—
Paired‐sample *t*‐test			
T0 vs. T1	40.83± 11.37	58.33	0.00
T0 vs. T2	46.96 ± 9.81	77.78	0.00
T1 vs. T2	6.13 ± 2.65	37.56	0.00
mRS‐pre vs mRS‐post	0.91 ± 0.81	12.52	0.032

Abbreviations: ICAS: intracranial atherosclerotic stenosis; mRS: modified Rankin Scale.

### 3.4 Risk Factor for Reduction of Stenosis Rate

Univariate linear regression analyses showed that age (*p* = 0.002) and smoking exposure (*p* = 0.034) produced a different response in terms of reduction of stenosis rate after intracranial stenting with the Neuroform Atlas stent (T0–T2). On the other hand, sex (*p* = 0.259), hypertension (*p* = 0.271), dyslipidemia (*p* = 0.419), diabetes (*p* = 0.237), alcoholism (*p* = 0.589), target artery (*p* = 0.586), and qualifying ischemic event (*p* = 0.352) did not. Multivariate linear regression analyses showed that age (*p* = 0.004) and smoking exposure (*p* = 0.04) were independent risk factors causing a different response in terms of the reduction of stenosis rate after intracranial stenting with the Neuroform Atlas stent (T0–T2). (Table 4).

**TABLE 4:** Results of univariate and multivariate linear regression analyses between total percentage reduction and various risk factors (N = 264).

Variables		Univariate				Multivariate					
		*n*	Mean (T0–T2)	B/t/RSR	*p*	B	β	t	*p*	*F*	Adjusted *R* ^2^
Age		264	46.96 ± 13.19	0.166	0.002^a^	0.156	0.177	2.925	0.004	7.002	0.044
Smoking exposure	Current	104	44.66 ± 9.82	115.97	0.034^b^	−1.319	−0.125	−2.061	0.04		
	Former	36	45.73 ± 9.79	140.32							
	Never	124	48.64 ± 9.61	145.26							
Sex	Male	164	47.76 ± 9.34	1.72	0.259^c^						
	Female	100	45.63 ± 10.45								
Hypertension	Yes	202	47.39 ± 10.08	1.30	0.271^c^						
	No	62	45.53 ± 8.81								
Dyslipidemia	Yes	217	46.76 ± 9.94	−0.694	0.419^c^						
	No	47	47.85 ± 9.20								
Diabetes	Yes	160	46.56 ± 9.98	−0.798	0.237^c^						
	No	104	47.55 ± 9.56								
Alcoholism	Current	86	47.40 ± 9.72	137.09	0.589^b^						
	Former	35	45.59 ± 11.02	121.33							
	Never	143	47.01 ± 9.59	137.09							
Target artery	ICA	10	47.05 ± 7.20	129.40	0.586^b^						
	MCA	109	46.20 ± 9.73	126.30							
	BA	82	48.18 ± 10.05	141.71							
	VA	63	46.64 ± 10.02	131.72							
Qualifying ischemic events	IS	189	46.27 ± 10.05	−1.806	0.352^c^						
	TIA	75	48.67 ± 9.00								

Abbreviations: BA: basilar artery; ICA: internal carotid artery; IS: ischemic stroke; MCA: middle cerebral artery; TIA: transient ischemic attacks; VA: vertebral artery.

^a^ Unitary linear regression analysis

^b^ Kruskal–Wallis test

^c^ Independent‐sample *t*‐test; *α*<0.05.

## 4 Discussion

Intracranial atherosclerosis is one of the most prevalent causes of stroke globally, specifically in Asian populations. Distal hypoperfusion, arteriovenous embolism, and the expansion of atheromatous plaques into the multitude of perforating arteries are the three major causes of ICAS following stroke (Holmstedt, Turan, and Chimowitz 2013). In the United States and Asia, IS caused by ICAS accounts for 8%–10% and up to 33% of cases, respectively (Broderick et al. 1998; Sacco et al. 1995; Wong et al. 1998). In recent years, scholars have provided the scientific rationale for cerebral angioplasty and stenting as an alternative treatment option (Gorelick et al. 2008; Kasner et al. 2006; Meyers et al. 2009). Stent implantation may be advantageous in ICAS patients with a stenosis rate of 70%–99%; this is according to a matched comparison between medically treated patients in the Warfarin Aspirin Symptomatic Intracranial Disease (WASID) research and those who received stent treatment at the National Institutes of Health intracranial stent registry (Qureshi et al. 2009). The Neuroform Atlas stent system was originally intended to treat wide‐necked intracranial aneurysms through stent‐assisted coiling (Jankowitz et al. 2019). Nonetheless, it can also be used to treat ICAS owing to its peculiar technical features. In recent years, only Orazio et al. and Ellenbogen et al. employed the Neuroform Atlas stent alone or combined with the Neurospeed balloon to treat patients with ICAS, with satisfactory results of treatment (Buonomo et al. 2021; Ellenbogen et al. 2023). Nevertheless, the two studies were single‐center and only enrolled 10 and 18 cases, respectively, which are not sufficiently reliable. Furthermore, other two recent studies have been documented on intracranial stenting with the Neuroform Atlas stent in conjunction with the use of the Gateway balloon for the treatment of ICAS in AIS, specifically as a rescue treatment following failure of mechanical thrombectomy and as an alternative treatment for ICAS (Takayanagi et al. 2021; Yi et al. 2021). To our knowledge, the present study has the largest cohort of the studies of ICAS treated with Neuroform Atlas stents. We thus demonstrated optimal technical success of endovascular treatment of 264 patients with sICAS using the Neuroform Atlas stent in our retrospective multi‐center study. We opted to use the Neuroform Atlas stent due to its several properties that enable endovascular treatment of ICAS with an excellent safety and efficacy profile.

Intracranial stenting with the Neuroform Atlas stent is an effective strategy to improve the underlying stenosis and relieve ischemic symptoms in patients with sICAS who have not responded to aggressive medical management. Herein, intracranial stenting with the Neuroform Atlas stent was performed on 264 patients with stenosis rates of 70%–99% in single‐vessel. Consequently, 226 patients had a good outcome (mRS 0–2) with no increase in target vessel ischemic area, 32 patients had a good outcome (mRS 0–2) but with stroke recurrence, and six patients experienced a 12‐month negative outcome (mRS 3–5). The number of patients with an mRS 0–2 was significantly higher after intracranial stenting than before intracranial stenting, with the paired‐sample *t*‐test showing a statistically significant difference between mRS‐pre and mRS‐post (*p* = 0.032). We found a statistically significant difference in the ICAS percentage in the target vessel at T1 compared to T0 (*p* = 0.00), as was the case for T2 compared with T0 (*p* = 0.00). These findings strongly suggest that intracranial stenting with the Neuroform Atlas stent for the treatment of symptomatic ICAS can effectively improve the underlying stenosis and relieve ischemic symptoms. Additionally, the significance of the ICAS percentage reduction between time points T0 and T1 and between time points T0 and T2 (T1 vs T2, *p* = 0.00) suggests that intracranial stenting with the Neuroform Atlas stent for the treatment of ICAS may help patients in both acute and chronic stages; this is due to the greater radial force of the Neuroform Atlas stent (Machi et al. 2017).

Intracranial stenting with the Neuroform Atlas stent is advantageous for treating sICAS, as it offers techniques unavailable with other stent systems. This is particularly beneficial when the target arteries are small, such as the MCA, BA and VA. The major constraint on the Wingspan stent is the intracranial manoeuvre needed to exchange the wire with a longer one (300 cm, 0.014 inch). This procedure has been involved in the increased incidence of ipsilateral cerebral hemorrhage noted in the SAMMPRIS study (Ellenbogen et al. 2023). Nonetheless, the Neuroform Atlas stent does not have a distal guide and can be delivered in a low‐profile microcatheter (0.014 inch). These characteristics could be extremely useful in the treatment of ICAS; they help in ensuring safe stent placement and minimizing the risk of iatrogenic damage, considering the uncertain clear visualization of the distal target vessel (e.g., preocclusive stenosis). As we know, the Wingspan stent has greater radial force and, in theory, is more effective at dilating atherosclerotic stenoses than the Neuroform Atals stent. However, placing the Wingspan stent is more difficult and laborious than placing the Neuroform Atals stent, which could lead to complications and reduce the surgical benefit to the patient. The proximal and distal markers of the Neuroform Atals stent are radiopaque inside the microcatheter, allowing good deployment of the stent, which remains open in the vessel, without shortening or lengthening. In this study, intracranial stenting with the Neuroform Atlas stent for sICAS had a complication rate of 6.06% (16/264), and the 6‐month ISR rate (stenosis rate≥70%) of 1.89% (5/264), which are lower than those reported in the WEAVE/WOVEN trial (Alexander et al. 2019, 2021). Possible reasons may include intracranial stenting with the Neuroform Atlas stent for the treatment of sICAS, eliminating the need for intracranial exchange manoeuvres and significantly reducing the risk of complications. This is achieved using an over‐the‐wire balloon, such as the Gateway balloon or the Neurospeed balloon catheter.

In addition, the Neuroform Atlas stent, with its cellular configuration, helps in proper apposition to the vessel walls and provides a low metal burden in the arterial space, making ISR unlikely (Buonomo et al. 2021). The two patients with postoperative complications of cerebral hemorrhage were stenosis of ICA and MCA, respectively, primarily due to hyperperfusion hemorrhage in the area of old cerebral infarction. The three patients with postoperative complications of cerebral infarction were stenosis of BA or VA, which were considered new‐onset cerebral infarcts majorly due to the disrupted flow or occlusion of the penetrating arteries of the target artery (vascular dissection). Summarily, in patients whose target artery is the anterior circulation, we believe that maintaining a stable systolic pressure (not exceeding 140 mmHg) before, during, and after intracranial stenting procedure is key to minimizing complications associated with intraoperative or postoperative hemorrhagic hyperperfusion cerebral hemorrhage. For patients with a target artery in the posterior circulation, intraoperative care by the operator to ensure the microguidewire remains within the true lumen of the target artery, as well as adequate fluid resuscitation during and after the procedure, are important in minimising complications related to cerebral infarction associated with “branch‐penetrating events.”

Furthermore, age and smoking exposure were independent risk factors that caused a different response in terms of reduction of stenosis rate in ICAS (T0–T2) after intracranial stenting with the Neuroform Atlas stent. The adjusted *R* ^2^ value of the model was 0.044, suggesting that age and smoking exposure explained 4.4% of the variation in the rate of improvement in arterial stenosis. The results of univariate linear regression analyses revealed that sex, hypertension, dyslipidemia, diabetes, alcoholism, target artery, and qualifying ischemic event did not cause a different response in terms of total percentage reduction in ICAS (T0–T2).

Additionally, pre‐operative VW‐HRMRI examination of patients may well help the operator to assess the luminal condition of the target artery and reduce inadvertent vascular injury at the same time improving the success rate of performing intracranial stenting. VW‐HRMRI can precisely assess plaque burden and provide information on plaque components, inflammation, and angiogenesis (Saam et al. 2007), of which are characteristics that help clinicians assess plaque vulnerability and predict future cerebrovascular events (Hosseini et al. 2013).

## 5 Limitations

This study has compelling shortcomings. First, its findings are based on a specific patient population and two particular hospitals, which might limit the generalizability of the results. Future research should therefore adopt more centers and randomly select sICAS patients from other centers. Second, we only enrolled stented patients with no control group; therefore, a comparative analysis would provide a better context for assessing the efficacy and safety of this particular stent. Third, this work is a retrospective observational cohort study, causing inevitable during the follow‐up.

## 6 Conclusion

Intracranial stenting with the Neuroform Atlas stent for sICAS is a potentially safe and effective procedure, with a statistically significant difference in the caliber of the affected vessel before and after treatment as well as a significant improvement in cerebral ischemic symptoms of patients. Larger prospective cohorts are essential in comprehensively assessing this stent for treating sICAS and validating the findings of this retrospective analysis.

## Author Contributions

**Weicheng Peng**: writing – review and editing, writing – original draft, methodology, conceptualization, visualization. **Haiyang Ma**: writing – original draft, writing – review and editing, methodology, visualization. **Shuaibin Lu**: software, data curation. **Xinli Xiang**: data curation, software. **Rui Zhao**: software, data curation. **Sheng Xu**: software, data curation. **Xupeng Peng**: software, data curation. **Yuhua Jiang**: conceptualization, investigation. **Zhiqiang Hu**: conceptualization, writing – review and editing, writing – original draft, supervision, formal analysis, methodology. **Feng Guan**: conceptualization, methodology, funding acquisition, writing – original draft, writing – review and editing, supervision, formal analysis, project administration.

## Ethics Statement

This multi‐center, retrospective study was conducted in accordance with the tenets of the Helsinki Declaration of 1975 as revised in 2000. The study was approved by the Institutional Review Board of Beijing Shijitan Hospital, Capital Medical University (Approval No.sjtkyll‐lx‐2022‐52), and Beijing Tiantan Hospital, Capital Medical University (Approval No.KY2023‐289‐02). All patients or their legal guardians in this study authorized the release of their medical records and information.

## Conflicts of Interest

The authors declare no conflicts of interest.

## Peer Review

The peer review history for this article is available at https://publons.com/publon/10.1002/brb3.70798

## Data Availability

The data presented in this study are available in article and supplementary material. Further inquiries can be directed to the corresponding authors.

## References

[brb370798-bib-0001] Alexander, M. J. , A. Zauner , J. C. Chaloupka , et al. 2019. “WEAVE Trial: Final Results in 152 on‐Label Patients.” Stroke; A Journal of Cerebral Circulation 50, no. 4: 889–894. 10.1161/STROKEAHA.118.023996. 31125298

[brb370798-bib-0002] Alexander, M. J. , A. Zauner , R. Gupta , et al. 2021. “The WOVEN Trial: Wingspan One‐Year Vascular Events and Neurologic Outcomes.” Journal of Neurointerventional Surgery 13, no. 4: 307–310. 10.1136/neurintsurg-2020-016208. 32561658

[brb370798-bib-0003] Broderick, J. , T. Brott , R. Kothari , et al. 1998. “The Greater Cincinnati/Northern Kentucky Stroke Study: Preliminary First‐Ever and Total Incidence Rates of Stroke Among Blacks.” Stroke; A Journal of Cerebral Circulation 29, no. 2: 415–421. https://pubmed.ncbi.nlm.nih.gov/9472883. 10.1161/01.str.29.2.415 9472883

[brb370798-bib-0004] Buonomo, O. , E. Mormina , A. A. Caragliano , et al. 2021. “Safety and Effect of Neuroform Atlas Stent in the Treatment of Symptomatic Intracranial Stenosis: A Single‐Center Experience.” Heliyon 7, no. 9: e08040. 10.1016/j.heliyon.2021.e08040. 34604563 PMC8473543

[brb370798-bib-0005] Coull, A. J. , and P. M. Rothwell . 2004. “Underestimation of the Early Risk of Recurrent Stroke: Evidence of the Need for a Standard Definition.” Stroke; A Journal of Cerebral Circulation 35, no. 8: 1925–1929. https://pubmed.ncbi.nlm.nih.gov/15192248. 10.1161/01.STR.0000133129.58126.67 15192248

[brb370798-bib-0006] Ellenbogen, Y. , E. J. Hendriks , S. Karadimas , et al. 2023. “Use of the Neuroform Atlas for Stenting of Intracranial Atherosclerotic Disease: Clinical and Angiographic Outcomes.” *Interventional Neuroradiology: Journal of Peritherapeutic Neuroradiology, Surgical Procedures and Related Neurosciences*. 10.1177/15910199231195134. PMC12852640 37817560

[brb370798-bib-0007] Elmadhoun, A. , H. Wang , and Y. Ding . 2024. “Impacts of Futile Reperfusion and Reperfusion Injury in Acute Ischemic Stroke.” Brain Circulation 10, no. 1: 1–4. 10.4103/bc.bc_9_24. 38655438 PMC11034445

[brb370798-bib-0008] GBD 2017 Causes of Death Collaborators . 2018. “Global, Regional, and National Age‐Sex‐Specific Mortality for 282 Causes of Death in 195 Countries and Territories, 1980–2017: A Systematic Analysis for the Global Burden of Disease Study 2017.” Lancet 392, no. 10159: 1736–1788. 10.1016/S0140-6736(18)32203-7. 30496103 PMC6227606

[brb370798-bib-0009] Gorelick, P. B. , K. S. Wong , H.‐J. Bae , and D. K. Pandey . 2008. “Large Artery Intracranial Occlusive Disease: A Large Worldwide Burden but a Relatively Neglected Frontier.” Stroke; A Journal of Cerebral Circulation 39, no. 8: 2396–2399. 10.1161/STROKEAHA.107.505776. 18535283

[brb370798-bib-0010] Holmstedt, C. A. , T. N. Turan , and M. I. Chimowitz . 2013. “Atherosclerotic Intracranial Arterial Stenosis: Risk Factors, Diagnosis, and Treatment.” The Lancet Neurology 12, no. 11: 1106–1114. 10.1016/S1474-4422(13)70195-9. 24135208 PMC4005874

[brb370798-bib-0011] Hosseini, A. A. , N. Kandiyil , S. T. S. Macsweeney , N. Altaf , and D. P. Auer . 2013. “Carotid Plaque Hemorrhage on Magnetic Resonance Imaging Strongly Predicts Recurrent Ischemia and Stroke.” Annals of Neurology 73, no. 6: 774–784. 10.1002/ana.23876. 23463579 PMC3824333

[brb370798-bib-0012] Jankowitz, B. T. , R. Hanel , A. P. Jadhav , et al. 2019. “Neuroform Atlas Stent System for the Treatment of Intracranial Aneurysm: Primary Results of the Atlas Humanitarian Device Exemption Cohort.” Journal of Neurointerventional Surgery 11, no. 8: 801–806. 10.1136/neurintsurg-2018-014455. 30670625 PMC6703120

[brb370798-bib-0013] Kasner, S. E. , M. I. Chimowitz , M. J. Lynn , et al. 2006. “Predictors of Ischemic Stroke in the Territory of a Symptomatic Intracranial Arterial Stenosis.” Circulation 113, no. 4: 555–563. https://pubmed.ncbi.nlm.nih.gov/16432056. 16432056 10.1161/CIRCULATIONAHA.105.578229

[brb370798-bib-0014] Krishnamurthi, R. V. , V. L. Feigin , M. H. Forouzanfar , et al. 2013. “Global and Regional Burden of First‐Ever Ischaemic and Haemorrhagic Stroke During 1990–2010: Findings From the Global Burden of Disease Study 2010.” The Lancet Global Health 1, no. 5: e259–e281. 10.1016/S2214-109X(13)70089-5. 25104492 PMC4181351

[brb370798-bib-0015] Machi, P. , F. Jourdan , D. Ambard , et al. 2017. “Experimental Evaluation of Stent Retrievers' Mechanical Properties and Effectiveness.” Journal of Neurointerventional Surgery 9, no. 3: 257–263. 10.1136/neurintsurg-2015-012213. 27016318 PMC5339553

[brb370798-bib-0016] Mazighi, M. , R. Tanasescu , X. Ducrocq , et al. 2006. “Prospective Study of Symptomatic Atherothrombotic Intracranial Stenoses: The GESICA Study.” Neurology 66, no. 8: 1187–1191. https://pubmed.ncbi.nlm.nih.gov/16636236. 16636236 10.1212/01.wnl.0000208404.94585.b2

[brb370798-bib-0017] Meyers, P. M. , H. C. Schumacher , R. T. Higashida , et al. 2009. “Indications for the Performance of Intracranial Endovascular Neurointerventional Procedures: A Scientific Statement From the American Heart Association Council on Cardiovascular Radiology and Intervention, Stroke Council, Council on Cardiovascular Surgery and Anesthesia, Interdisciplinary Council on Peripheral Vascular Disease, and Interdisciplinary Council on Quality of Care and Outcomes Research.” Circulation 119, no. 16: 2235–2249. 10.1161/CIRCULATIONAHA.109.192217. 19349327

[brb370798-bib-0018] Miao, Z. , Y. Zhang , J. Shuai , et al. 2015. “Thirty‐Day Outcome of a Multicenter Registry Study of Stenting for Symptomatic Intracranial Artery Stenosis in China.” Stroke; A Journal of Cerebral Circulation 46, no. 10: 2822–2829. 10.1161/STROKEAHA.115.010549. 26286544

[brb370798-bib-0019] Park, S.‐Y. , J.‐S. Oh , H.‐J. Oh , S.‐M. Yoon , and H.‐G. Bae . 2017. “Safety and Efficacy of Low‐Profile, Self‐Expandable Stents for Treatment of Intracranial Aneurysms: Initial and Midterm Results—A Systematic Review and Meta‐Analysis.” Interventional Neurology 6, no. 3–4: 170–182. 10.1159/000471890. 29118794 PMC5662986

[brb370798-bib-0020] Qureshi, A. I. , E. Feldmann , C. R. Gomez , et al. 2009. “Consensus Conference on Intracranial Atherosclerotic Disease: Rationale, Methodology, and Results.” Journal of Neuroimaging: Official Journal of the American Society of Neuroimaging 19, no. Suppl 1: 1S–10S. 10.1111/j.1552-6569.2009.00414.x. 19807850

[brb370798-bib-0021] Saam, T. , T. S. Hatsukami , N. Takaya , et al. 2007. “The Vulnerable, or High‐Risk, Atherosclerotic Plaque: Noninvasive MR Imaging for Characterization and Assessment.” Radiology 244, no. 1: 64–77. https://pubmed.ncbi.nlm.nih.gov/17581895. 17581895 10.1148/radiol.2441051769

[brb370798-bib-0022] Sacco, R. L. , D. E. Kargman , Q. Gu , and M. C. Zamanillo . 1995. “Race‐Ethnicity and Determinants of Intracranial Atherosclerotic Cerebral Infarction. The Northern Manhattan Stroke Study.” Stroke; A Journal of Cerebral Circulation 26, no. 1: 14–20. https://pubmed.ncbi.nlm.nih.gov/7839388. 10.1161/01.str.26.1.14 7839388

[brb370798-bib-0023] Samuels, O. B. , G. J. Joseph , M. J. Lynn , H. A. Smith , and M. I. Chimowitz . 2000. “A Standardized Method for Measuring Intracranial Arterial Stenosis.” American Journal of Neuroradiology 21, no. 4: 643–646. https://pubmed.ncbi.nlm.nih.gov/10782772. 10782772 PMC7976653

[brb370798-bib-0024] Santillan, A. , S. Boddu , J. Schwarz , et al. 2018. “LVIS Jr. Stent for Treatment of Intracranial Aneurysms With Parent Vessel Diameter of 2.5 Mm or Less.” Interventional Neuroradiology: Journal of Peritherapeutic Neuroradiology, Surgical Procedures and Related Neurosciences 24, no. 3: 246–253. 10.1177/1591019918759307. 29463145 PMC5967194

[brb370798-bib-0025] Stracke, C. P. , L. Meyer , J. Fiehler , et al. 2020. “Intracranial Bailout Stenting With the Acclino (Flex) Stent/NeuroSpeed Balloon Catheter After Failed Thrombectomy in Acute Ischemic Stroke: A Multicenter Experience.” Journal of Neurointerventional Surgery 12, no. 1: 43–47. 10.1136/neurintsurg-2019-014957. 31239330

[brb370798-bib-0026] Suri, M. F. K. , Y. Qiao , X. Ma , et al. 2016. “Prevalence of Intracranial Atherosclerotic Stenosis Using High‐Resolution Magnetic Resonance Angiography in the General Population: The Atherosclerosis Risk in Communities Study.” Stroke; A Journal of Cerebral Circulation 47, no. 5: 1187–1193. 10.1161/STROKEAHA.115.011292. PMC5319392 27056984

[brb370798-bib-0027] Takayanagi, A. , P. K. Cheng , and L. Feng . 2021. “A Novel Technique for Stenting of Intracranial Stenosis Using the Neuroform Atlas Stent and Gateway Balloon Catheter.” Interventional Neuroradiology: Journal of Peritherapeutic Neuroradiology, Surgical Procedures and Related Neurosciences 27, no. 6: 770–773. 10.1177/15910199211007295. 33823620 PMC8673898

[brb370798-bib-0028] Wang, C. C. , W. Li , Z. Z. Feng , et al. 2017. “Preliminary Experience With Stent‐Assisted Coiling of Aneurysms Arising From Small (<2.5 mm) Cerebral Vessels Using the Low‐Profile Visualized Intraluminal Support Device.” American Journal of Neuroradiology 38, no. 6: 1163–1168. 10.3174/ajnr.A5145. 28385886 PMC7960081

[brb370798-bib-0029] Wong, K. S. , Y. N. Huang , S. Gao , W. W. Lam , Y. L. Chan , and R. Kay . 1998. “Intracranial Stenosis in Chinese Patients With Acute Stroke.” Neurology 50, no. 3: 812–813. https://pubmed.ncbi.nlm.nih.gov/9521286. 9521286 10.1212/wnl.50.3.812

[brb370798-bib-0030] Yang, Y. , Z. Wang , Q. Hu , L. Liu , G. Ma , and C. Yang . 2024. “Enhancing the Clinical Value of Single‐Phase Computed Tomography Angiography in the Assessment of Collateral Circulation in Acute Ischemic Stroke: A Narrative Review.” Brain Circulation 10, no. 1: 35–41. 10.4103/bc.bc_54_23. 38655435 PMC11034444

[brb370798-bib-0031] Yi, H. J. , J. H. Sung , and D. H. Lee . 2021. “Preliminary Experience of Neuroform Atlas Stenting as a Rescue Treatment After Failure of Mechanical Thrombectomy Caused by Residual Intracranial Atherosclerotic Stenosis.” Journal of Korean Neurosurgical Society 64, no. 2: 198–206. 10.3340/jkns.2020.0146. 33715323 PMC7969043

[brb370798-bib-0032] Zaidat, O. O. , R. A. Hanel , E. A. Sauvageau , et al. 2020. “Pivotal Trial of the Neuroform Atlas Stent for Treatment of Anterior Circulation Aneurysms: One‐Year Outcomes.” Stroke; A Journal of Cerebral Circulation 51, no. 7: 2087–2094. 10.1161/STROKEAHA.119.028418. PMC7306258 32568654

[brb370798-bib-0033] Zhang, W. , Y. Li , M. Pang , and X. Yue . 2024. “Causal Relationship Between Hypertension and Ischemic Stroke: a Two‐Sample Mendelian Randomization Study.” Brain Circulation 10, no. 3: 257–264. 10.4103/bc.bc_105_23. 39526106 PMC11542756

[brb370798-bib-0034] Zhao, M. , Y. Qiao , A. Weiss , and W. Zhao . 2024. “Neuroprotective Strategies in Acute Ischemic Stroke: A Narrative Review of Recent Advances and Clinical Outcomes.” Brain Circulation 10, no. 4: 296–302. 10.4103/bc.bc_165_24. 40012592 PMC11850939

[brb370798-bib-0035] Zhou, M. , H. Wang , J. Zhu , et al. 2016. “Cause‐Specific Mortality for 240 Causes in China During 1990–2013: A Systematic Subnational Analysis for the Global Burden of Disease Study 2013.” Lancet 387, no. 10015: 251–272. 10.1016/S0140-6736(15)00551-6. 26510778

